# Conservative Management of Coronary Artery Disease With Ectasia in Antiphospholipid Syndrome-Associated Thrombocytopenia: A Case Report

**DOI:** 10.7759/cureus.90786

**Published:** 2025-08-23

**Authors:** Marha M Menaisy, Karim Shahin, Hager M Kamal, Kareem Elkharashy, Ali A Elzieny

**Affiliations:** 1 Department of Cardiology and Angiology, Kasr AlAiny Faculty of Medicine, Cairo University, Cairo, EGY; 2 Department of Critical Care Medicine, Faculty of Medicine, Alexandria University, Alexandria, EGY; 3 Department of Cardiology and Angiology, Alexandria Main University Hospital, Alexandria, EGY; 4 Department of Internal Medicine, Faculty of Medicine, Alexandria University, Alexandria, EGY; 5 Department of Radiology, Boston University Chobanian & Avedisian School of Medicine, Boston, USA

**Keywords:** acute coronary syndrome, antiphospholipid antibody syndrome (aps), coronary artery ectasia (cae), coronary catheterization, secondary immune thrombocytopenia

## Abstract

Despite the fact that cardiovascular involvement in antiphospholipid syndrome (APS) is less common, it carries serious complications, especially in preexisting coronary artery disease (CAD) and ectasia. We present a 55-year-old man diagnosed with APS aggravated by secondary immune thrombocytopenic purpura. One year later, he presented with an acute myocardial infarction in the context of thrombocytopenia, which was challenging. A coronary angiogram revealed a total distal left anterior descending artery occlusion and coronary artery ectasia with diffuse atherosclerosis. Conservative management was chosen due to lower procedural success and higher incidence of complications, and his follow-up showed no functional impairment (stationary left ventricular ejection fraction) and compliance with heart-remodifying medications. Thus, the management of CAD in such patients remains very challenging and requires cautious treatment.

## Introduction

Antiphospholipid syndrome (APS) is a multisystem autoimmune disorder characterized by hypercoagulability and vascular thrombosis in the presence of persistently elevated antiphospholipid antibodies (APLAs). APS can be generally classified as either primary or secondary based on whether there is an association with an underlying systemic autoimmune disease. In addition, the laboratory classification criteria for APS include lupus anticoagulant (LAC), anticardiolipin antibodies, and anti-B2 Glycoprotein (GP) immunoglobulin G (IgG) or immunoglobulin M (IgM), each of which has its significance in the manifesting presentation [1]. These antibodies have been found in about 5% of the healthy population. Despite that, not all patients with positive APLAs would develop APS [2].

The clinical manifestation of APS is mainly through the thrombotic mechanism, with venous thromboses being more common than arterial thromboses. According to large cohort studies, the most common location of thrombosis is the deep veins of the lower extremities, ranging from 20% to 30% of individuals with APS. In contrast, the cerebral vasculature is the most common site for arterial thrombosis (13% stroke and 7% transient ischemic attack) [3]. However, one-third of the patients with APS develop thrombocytopenia via a postulated mechanism of APS antibodies. Its sole effect was reported to have a minimal significance as it presents as mild and rarely causes major bleeding, yet if conjunctively associated with a secondary immune thrombocytopenic purpura (ITP), it would require cautious progression in management. This dual pathology complicates the disease course by being more severe and symptomatic [4].

Cardiovascular involvement is less common; however, it carries serious complications. Cardiac features of APS involve valvular heart disease, myocardial infarction (MI), and coronary artery disease (CAD). CAD may be the first presentation of APS; however, acute myocardial infarction (AMI) in patients with APS usually occurs in the fourth decade of life [5]. A review by Chighizola et al. found that a two-fold increased risk of MI was associated with APLAs (including anticardiolipin anti-eta 2 GP 1 and LAC antibodies) [6]. Among MI patients, APS is not the sole culprit, especially when the patient has other significant associated risk factors such as hyperlipidemia, hyperglycemia, smoking, and other preliminary cardiac conditions such as coronary artery ectasia (CAE) [7]. Such risk factors would highly impact thrombotic formation in APS patients. Managing such patients is complex and requires multidisciplinary care.

Herein, we describe the importance of bringing a multidisciplinary team together to postulate an individualized management plan for a clinically challenging scenario of our patient who developed an AMI associated with CAE complicating APS with secondary ITP.

## Case presentation

A 55-year-old man, who was physically active and a heavy smoker, sought medical attention in the Hematology clinic after experiencing substantial ear bleeding, severe purpura, and numerous ecchymotic patches on both lower limbs. The patient had a history of prior deep venous thrombosis (DVT) five years ago and was on warfarin for six months. Laboratory results (Table 1) showed low platelet count (14 × 10^3^/uL), normal values for coagulation and bleeding profiles, and a negative virology screening.

**Table 1:** Laboratory results of a patient with antiphospholipid syndrome associated with secondary immune thrombocytopenia at the initial presentation

Laboratory investigation	Result	Reference range
Hemoglobin	13.5 g/dL	13-17 g/dL
White blood count	8.5 × 10^3^/uL	4-10 × 10^3^/uL
Platelets count	14 × 10^3^/uL	150-410 × 10^3^/uL
Prothrombin time	13.0 seconds	11-14 seconds
Activated partial thromboplastin time	43.2 seconds	26-40 seconds
International normalized ratio	1.1	Up to 1.2
Human immunodeficiency virus	0.11 nonreactive	Negative
Hepatitis B surface antigen	0.19 nonreactive	Negative <1
Hepatitis C antibody	0.11 nonreactive	Negative <1
Antinucleotide-antibody titer and pattern	1/160 cu	<60 years; <1/40 cu
Anti-double-stranded deoxyribonucleic acid antibodies	<9.8 U/mL	0-35 U/mL
Lupus anticoagulant	80 g/L	20-39 g/L
Anticardiolipin immunoglobulin M	169.6 U/mL	0-30 U/mL
Beta-2 glycoprotein immunoglobulin G	26.3 cu	0-20 cu

Furthermore, the immunological factor survey revealed positive results for the antinuclear antibody (titer 1/160 cu homogenous), LAC, anticardiolipin IgM, and anti-B2 GP IgG with persistent elevation. However, the anti-ds-DNA antibody was negative. An ultrasound of the abdomen revealed no organomegaly. Lower limb Doppler studies revealed no recent DVT while detecting diffuse arterial atherosclerotic changes with scattered tiny calcified atheromatous plaques. The patient was admitted to the hematology unit after being given the diagnosis of APS associated with secondary ITP. He received high-dose steroid medication in the form of methylprednisolone pulse therapy, and subsequently administered cyclosporin. The bleeding has stopped, and the platelet count has improved during the hospital stay. The patient was discharged on oral therapy with a recommendation to return to the outpatient clinic for a follow-up.

**Figure 1:**
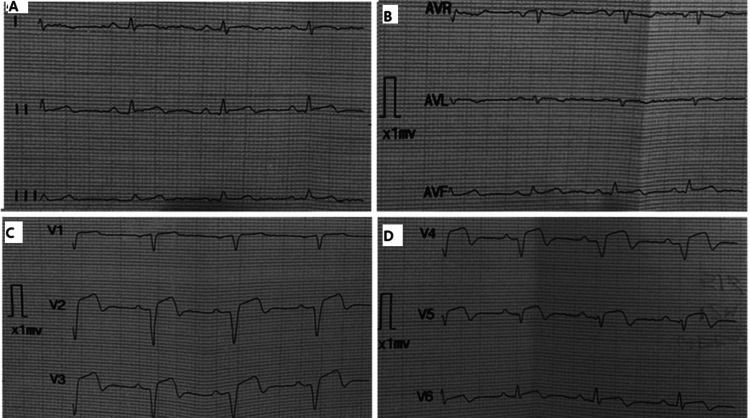
Electrocardiogram (A,B) limb leads and (C,D) chest leads of a patient with antiphospholipid syndrome diagnosed with acute myocardial infarction showing ST-segment elevation in the precordial leads (V1-6) with subsequent Q-wave development AVR: augmented vector right; AVF: augmented vector foot; AVL: augmented vector left

**Figure 2:**
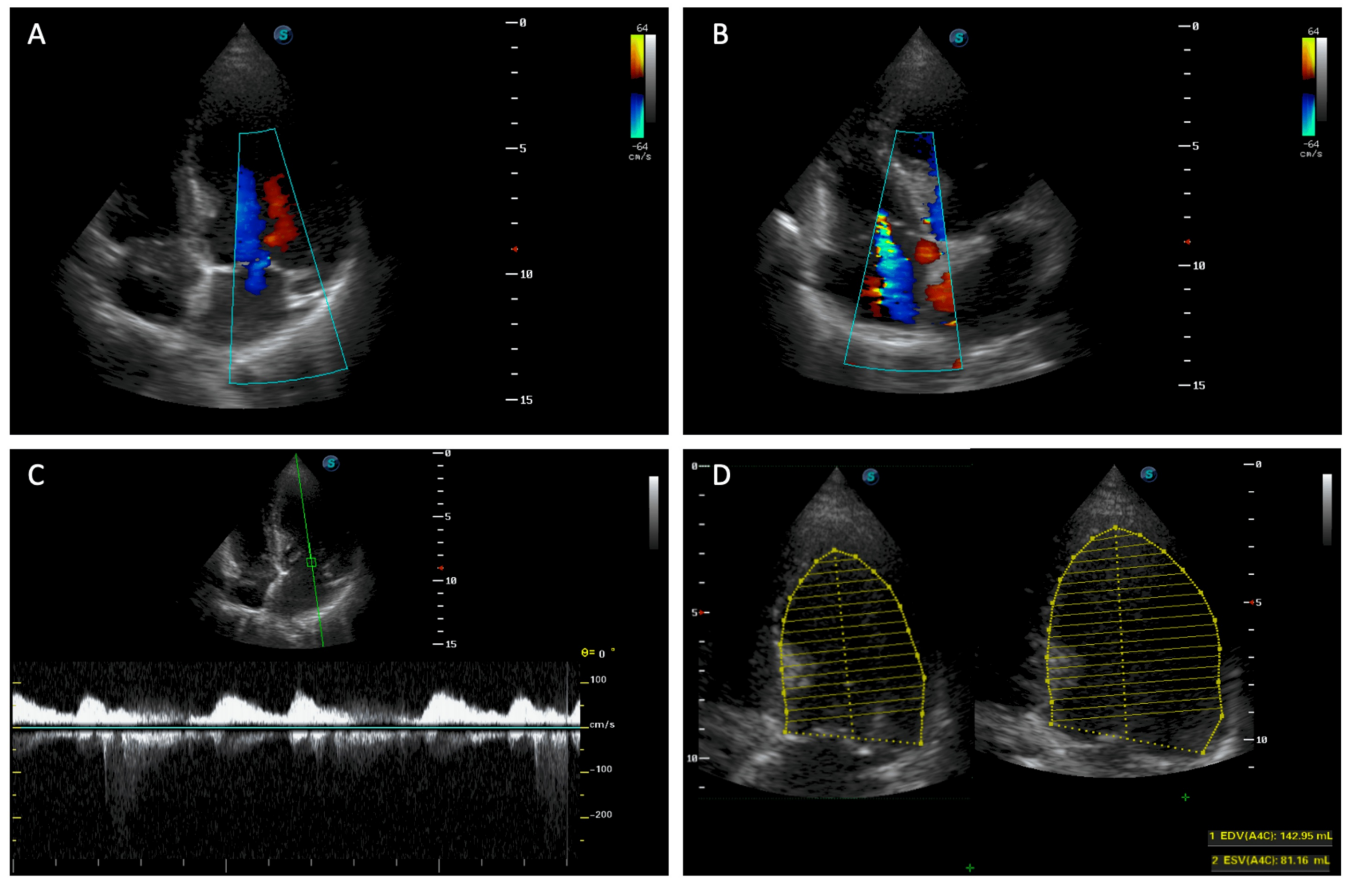
Transthoracic echocardiogram of a patient with antiphospholipid syndrome diagnosed with acute myocardial infarction showing an apical four-chamber view with color Doppler over mitral and tricuspid valves, reveling mild mitral regurgitation (A) and moderate tricuspid regurgitation (B), spectral Doppler flow over the mitral valve (C), and calculation of left ventricle systolic function using modified Simpson technique (D)

**Figure 3:**
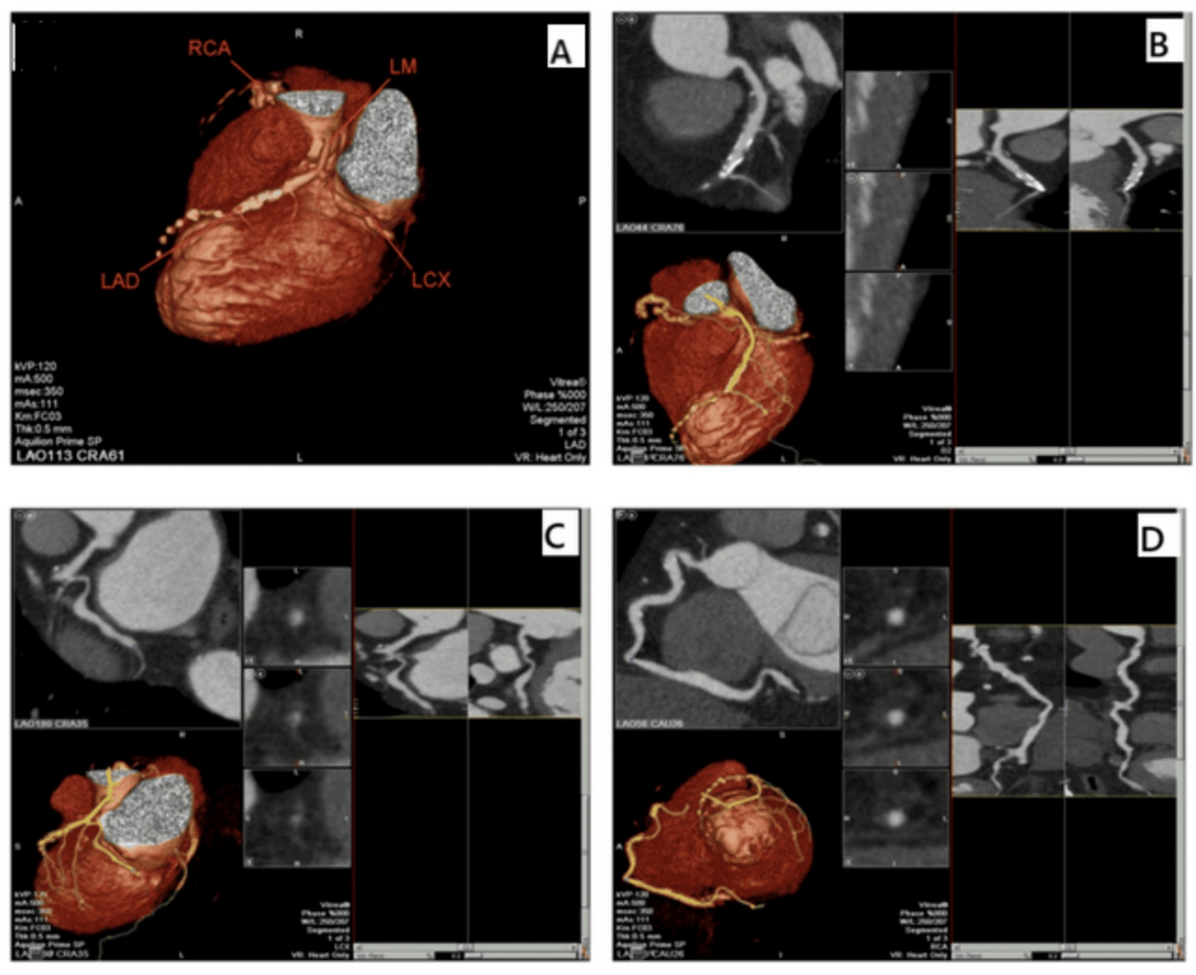
Computed tomography coronary angiogram of a patient with antiphospholipid syndrome diagnosed with acute myocardial infarction revealing (A) heart volume rendering with left and right coronary systems, (B) distal total occlusion of the LAD artery with mid-segment nonobstructed mixed plaques and severe calcium accumulation, (C) ectatic LCX with nonobstructive mixed plaques, (D) ectatic right coronary artery with nonobstructive mixed plaques and scattered severe coronary calcium deposition LM: left main; LAD: left anterior descending; RCA: right coronary artery; LCX: left circumflex artery

One year later, the patient presented to the emergency room with acute severe chest discomfort radiating to the back and the left shoulder that was accompanied by cold sweats and vomiting. The initial electrocardiogram showed ST-segment elevation in the pericardial leads (V1-6) with subsequent Q wave development (Figure 1). Per a heart team discussion, it was decided that the patient was at high risk for percutaneous coronary intervention based on a low platelet count follow-up (40 × 10^3^/uL) the day before presentation. The decision was made by the heart team according to the local protocol of managing ST-elevation myocardial infarction in severe thrombocytopenic patients. Thus, the patient was admitted to the Cardiac Care Unit and given thrombolytic therapy with streptokinase with close monitoring over several days until he was hemodynamically stable. Echocardiography showed reduced left ventricular (LV) systolic function along with regional wall motion abnormalities, including apical cap dyskinesia, anteroseptal akinesia, and anterior wall hypokinesia (Figure 2). To determine the true anatomy of the coronaries in the setting of low platelet counts and high bleeding risk, computed tomography coronary angiogram (CTCA) was ordered, which revealed distal total occlusion of the left anterior descending (LAD) artery, ectatic right coronary artery (RCA) and left circumflex artery (LCX), nonobstructive mixed plaques in the right and left systems, and severe coronary calcium accumulation with globally poor LV function (Figure 3).

**Figure 4:**
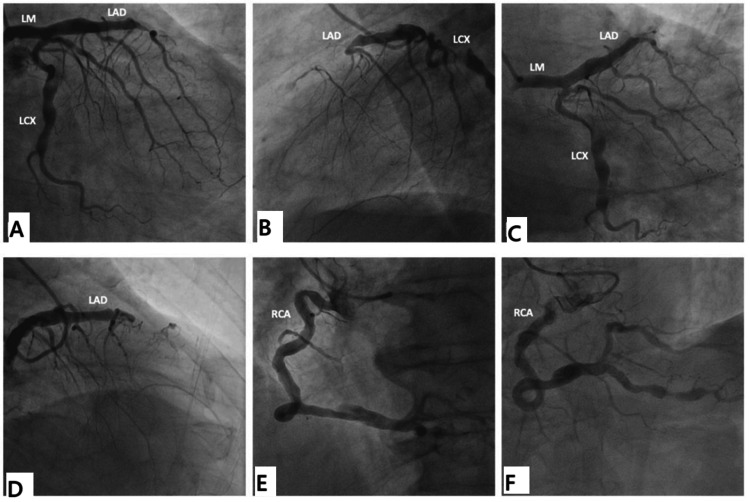
Coronary angiography was performed on a patient with antiphospholipid syndrome diagnosed with acute myocardial infarction after stabilizing his condition and raising his platelet counts with pulse steroid combined with rituximab over three weeks. (A) RAO18.36°/Caudal22.13°, (B) LAO90.1°/Cranial0.6°, (C) RAO 0.82°/Caudal38.8°, and (D) RAO19.8°/Cranial19.8° show a normal left main that bifurcates into ectatic both LAD and LCX with total distal LAD occlusion that filled retrograde. (E) LAO 34.4°/Caudal1.3° and (F) RAO0.6°/Cranial24.4° show ectatic RCA LM: left main; LAD: left anterior descending; RCA: right coronary artery; LCX: left circumflex artery

Following the CTCA findings, a multidisciplinary team was assembled, including an interventional cardiologist and hematologist, to approach the case in an individualized manner. The interventional cardiologist highlighted the importance of coronary angiography for better delineation of the preexisting ectatic lesions. However, the patient’s low platelet count puts him at high risk of bleeding. Hence, the hematological team opted to intensify the treatment in the form of pulse steroid combined with rituximab, which demonstrated a rise in the platelet counts to 100 × 10^3^/uL after three doses over three weeks. The heart team simultaneously planned to perform a dobutamine stress echocardiography to evaluate the myocardial viability, which revealed viable anterior and anteroseptal walls with a nonviable apex at low-dose dobutamine. Afterward, a coronary angiography was performed, showing a normal left main that bifurcates into ectatic both LAD and LCX with total distal LAD occlusion that filled retrograde and ectatic RCA (Figure 4).

**Figure 5:**
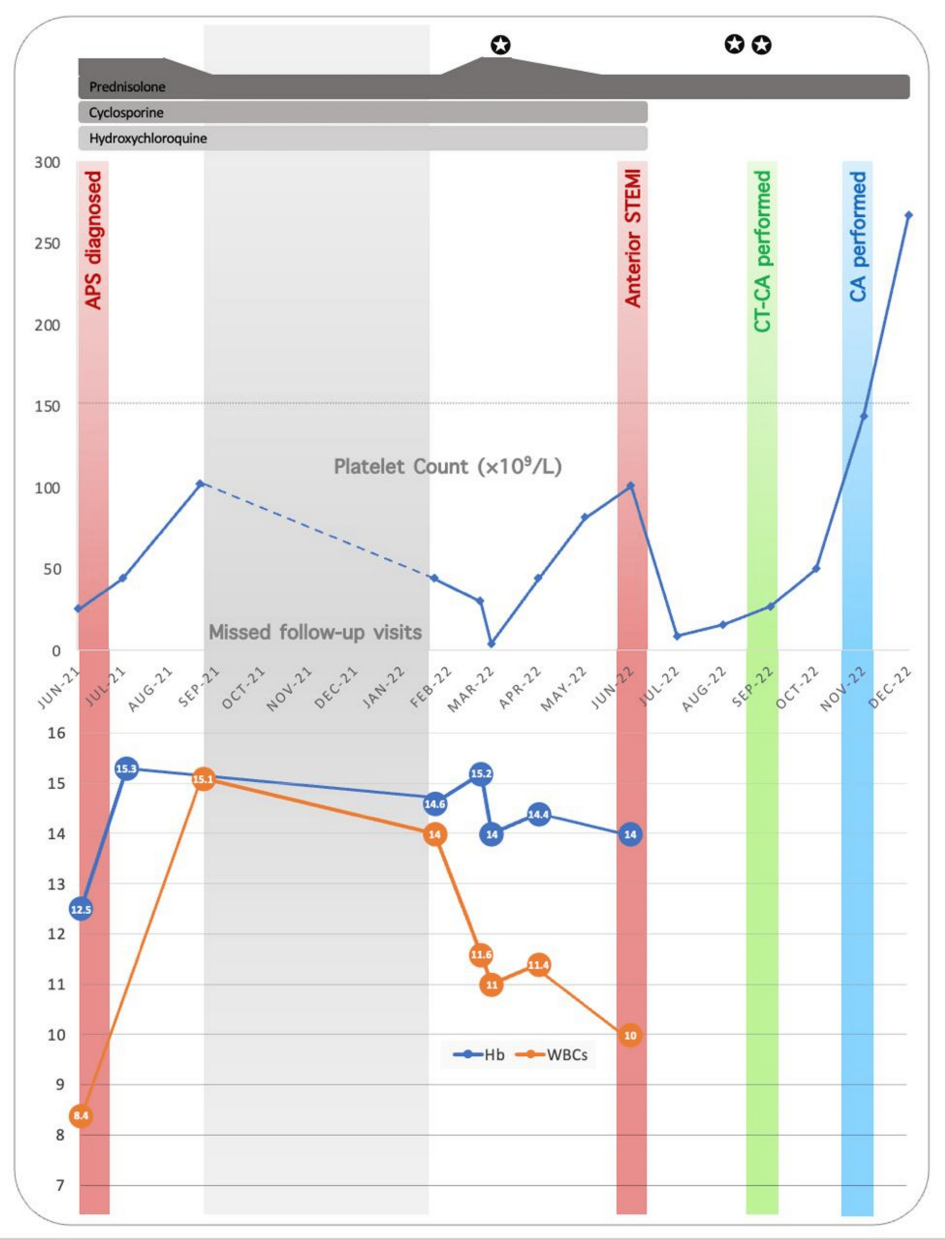
Changes in platelet count in a patient with APS and secondary immune thrombocytopenia. The time course for prednisolone, cyclosporine, and hydroxychloroquine is shown in the upper portion of the graph. The rounded star indicates time points of dexamethasone administration APS: antiphospholipid syndrome; CT-CA: computed tomography coronary angiogram; Hb: hemoglobin; WBCs: white blood cells; STEMI: ST-elevation myocardial infarction

The decision for no intervention was agreed upon by the interventional cardiologists due to concerns that it would be associated with lower procedural success and a higher incidence of complications. After a multidisciplinary team discussion, the patient was discharged with a regimen of warfarin, bisoprolol, ramipril, pantoprazole, atorvastatin, and low-dose prednisone with azathioprine. Weekly follow-up in outpatient clinics was advised. Figure 5 shows platelet count changes throughout the patient’s medical events.

During the following six months, a multidisciplinary outpatient team followed up and monitored the patient clinically to allow an uptitration of the anticoagulant until reaching the targeted INR (2-3) and heart remodifying medications to the maximum tolerating doses, permitting the best quality of life for the patient. He reported no significant functional impairment or hospital readmission during this duration. His follow-up labs showed that platelet count was maintained above 100 × 10^3^/uL with unremarkable other tests. His echocardiogram showed moderately dilated left ventricular internal dimensions with reduced ejection fraction without aneurysmal dilatation or LV apical thrombus. The patient was satisfied with the treatment plan and management approach.

## Discussion

Cardiovascular complications contribute significantly to morbidity and mortality in patients with APS, including accelerated atherosclerosis, acute coronary syndrome (ACS), thrombi (venous, arterial, or intracardiac), marantic endocarditis, and cardiomyopathy. Yet, ACS is the most serious complication that was reported in patients with APS and may be the first presentation. Interestingly, coronary embolism should be considered in ACS etiology as one of the contributing factors, given the classical atherothrombotic mechanisms in APS patients through the molecular effect of its antibodies on endothelial cells that increase the risk of CAD development [8]. Indeed, ACS often occurs in the absence of significant epicardial coronary artery stenosis. Furthermore, postprocedural complications occur frequently with ACS. However, the specific management of these complications is not well documented for such patients. Thus, the management of cardiac complications in APS patients remains challenging. Our case emphasizes the individualized multidisciplinary management of such scenarios.

CAE is the dilatation of an arterial segment to a diameter at least 1.5 times that of the adjacent normal artery. It is attributed to atherosclerosis in 50% of cases. However, inflammatory or connective tissue disease was associated with CAEs in 10%-20%. A sluggish or turbulent blood flow induced by the presence of ectatic segments was accompanied by an increased incidence of chest pain and MI, regardless of the severity of coexisting stenotic lesions [9].

Prolonged anticoagulation as the main therapy was suggested by previous studies despite inadequately addressed medical management. Therefore, this treatment cannot be recommended until supported by further studies. On the other hand, patients with coexisting obstructive lesions benefit from restoring myocardial perfusion using either or both percutaneous and/or surgical coronary revascularization [10]. Unfortunately, there is a lack of literature regarding the proper management of CAE in patients with APS. In our case, we opted for conservative management due to a higher incidence of complications and a lower procedural success rate.

Cardiovascular risk stratification is essential in APS, given the burden of cardiovascular events and accelerated atherosclerosis in such patients. This facilitates implementing primary and secondary prevention strategies by healthcare providers [11]. Accordingly, a study assessed the effectiveness of the adjusted Global Antiphospholipid Syndrome Score, designed to estimate overall thrombotic complications and specifically predict cardiovascular disease [12]. As outlined in the recommendations of the 13th International Congress on Antiphospholipid Antibodies, APS patients should be categorized into high-risk and low-risk groups. The high-risk group encompasses individuals exhibiting multiple APLA positivity, LAC positivity, or persistent anticardiolipin positivity, along with the concomitant presence of autoimmune disease such as systemic lupus erythematosus or rheumatoid arthritis [11]. In addition, a prior history of thrombotic events and conventional cardiovascular risk factors should be considered in defining the high-risk group as per the European League Against Rheumatism [13].

Treatment of traditional cardiovascular risk factors is recommended for the primary prevention of APS as well as in the general population. Additionally, in high-risk-profile asymptomatic antibody carriers, it is recommended to give low-dose aspirin as primary prophylaxis, while it is considered individually for those with a low-risk profile. Anticoagulation therapy is the recommended treatment for thrombosis in APS patients, overlapping between a vitamin K antagonist (VKA) and unfractionated or low-molecular-weight heparin, with no current recommendation for direct oral anticoagulants. VKA treatment is the basis of secondary prevention targeting an international normalized ratio (INR) of 2-3. High-intensity VKA or combined therapy should be considered in cases of recurrent events despite adequate treatment, with an INR target of 3-4. To date, it is recommended to follow current guidelines for the management of ACS in the general population [11]. Our patient was discharged, and VKA treatment was monitored to a target INR between 2 and 3.

Our case highlights the importance of individualized management of challenging APS patients with acute MI associated with secondary ITP. Therefore, further studies or clinical guidelines tailored to APS patients with cardiovascular involvement and concurrent thrombocytopenia are needed.

## Conclusions

The multidisciplinary teams caring for patients with AMI must have a high degree of suspicion of causes other than traditional CAD. This case poses some significant points for the management of CAD with APS complicated by secondary ITP purpura, which are clinically challenging. Our case highlights the challenging management of such patients regarding noninvasive vs. invasive imaging and therapeutic approaches.
